# Occult Spinal Dysraphism in Obstetrics: A Case Report of Caesarean Section with Subarachnoid Anaesthesia after Remifentanil Intravenous Analgesia for Labour

**DOI:** 10.1155/2012/472482

**Published:** 2012-07-10

**Authors:** A. Valente, L. Frassanito, L. Natale, G. Draisci

**Affiliations:** ^1^Istituto di Anestesiologia e Rianimazione, Università Cattolica del Sacro Cuore, Largo A. Gemelli 8, 00168 Rome, Italy; ^2^Istituto di Radiologia, Università Cattolica del Sacro Cuore, Largo A. Gemelli 8, 00168 Rome, Italy

## Abstract

Neuraxial techniques of anaesthesia and analgesia are the current choice in obstetrics for efficacy and general low risk of major complications. Concern exists about neuraxial anaesthesia in patients with occult neural tube defects, regarding both labour analgesia and anaesthesia for Caesarean section. Recently, remifentanil infusion has been proposed as an analgesic technique alternative to lumbar epidural, especially when epidural analgesia appears to be contraindicated. Here, we discuss the case of a pregnant woman attending at our institution with occult, symptomatic spinal dysraphism who requested labour analgesia. She was selected for remifentanil intravenous infusion for labour pain and then underwent urgent operative delivery with spinal anaesthesia with no complications.

## 1. A Case Report

A 35-year-old woman in her first pregnancy came to us in week 38 of gestation for an ambulatory examination to make determinations about labour analgesia. She had a history of a congenital dysmorphic foot with previous orthopaedic surgery for correction and femur elongation. She reported no sensory deficits. The patient offered radiographic evidence of posterior schisis of all lumbar and sacral vertebrae, as is seen with spina bifida, with no signs of pelvic anomalies.

She could not offer any computed tomography or magnetic resonance imaging (MRI) images of neuraxis, and time before delivery was too short to program MRI execution. An epidural approach to intrapartum analgesia was then discouraged, and the patient was considered for intravenous remifentanil analgesia at the moment of labour.

The patient was admitted to our hospital for excess weight gain (55 to 75 kg weight, 160 cm height) in term pregnancy (40th week). Clinical conditions were good, and laboratory examinations showed no significant alterations. 

Because of oligohydramnios and advanced gestational age, labour was induced with dinoprostone vaginal gel 1 mg. During the night, 9 hours after dinoprostone application, the patient's cervix had dilated to 2 cm, and she was experiencing 5 uterine contractions/15 min with a mobile presented head. At this time, patient-controlled remifentanil infusion was started. 

We used a patient-controlled analgesia (PCA) Alaris syringe pump. Remifentanil dilution was 2 mg in 40 mL NaCl 0,9% solution (i.e., 50 *μ*g/mL). Bolus speed was 1 mL/sec. We used a separate intravenous infusion line for remifentanil, with rigid, thin tubing, so no fluid bolus was needed to flush any dead space. Patient electrocardiogram (ECG), peripheral oxygen saturation (SpO_2_), and noninvasive blood pressure (10-minute interval) were monitored. Oxygen was immediately available if needed.

Our remifentanil infusion protocol consists of a 0.025 *μ*g/kg/min continuous infusion with 2-minute interval access to patient-controlled boluses. The patient was instructed to call for bolus as she first felt a contraction. Boluses were initially of 0.25 *μ*g/kg and could be progressively increased to 0.375, 0.50, 0.625, and 0.750 *μ*g/kg if the visual analogue scale (VAS) was greater than 4 for more than three subsequent uterine contractions. 

Pain relief was satisfactory (i.e., VAS less than 4) for 8 h using incremental boluses up to 0.625 *μ*g/kg, allowing the patient to reach 8 cm cervical dilatation. Labour was augmented through oxytocin infusion (0,12–0,36 IU/h) by the gynaecologist.

After this time, pain control was not complete (VAS 7-8), and a further increase of bolus dose was not allowed because of patient sedation and reduced ability to control analgesic bolus timing.

About two hours later, and nineteen hours after vaginal gel application, delivery through caesarean section was indicated because of arrest of descent and asynclitism. Remifentanil infusion was then stopped. The option of subarachnoid anaesthesia was considered.

With the patient in the sitting position, the L4-L5 interspace was identified as the space inferior to iliac crest level, and a lumbar puncture was performed at this location through a 27 G 3.5-inch Whitacre spinal needle with an introducer. After the initial appearance of blood, clear cerebrospinal fluid flowed, and 15 *μ*g fentanyl and 10 mg 1% hyperbaric bupivacaine were injected through needle. The patient was then turned supine, with about 20° left uterine displacement. Oxygen 50% was delivered through a face mask to maximize arterial oxygen content.

Evidence of block onset was offered by the patient only after about 4 minutes, which required a slight shift to the Trendelenburg table position; anaesthesia reached approximately the T6 pin-prick level in about 14 minutes, at which point surgery was begun.

3600 grams for the weight of the baby was delivered 25 minutes after spinal anaesthesia execution. Apgar scores at 1 and 5 minutes were each 9. The surgery lasted about 60 minutes. No infant complications were noted.

At the end of intervention, partial motor recovery began and appeared to be complete in about 3 hours. Post-operative pain was controlled using 10 mg s.c. morphine at the end of intervention and 90 mg i.v. ketorolac in the first 24 hours. The patient tried a sitting position on the morning after surgery and walked after a few hours, with no appearance of headache.

**Figure 1:**
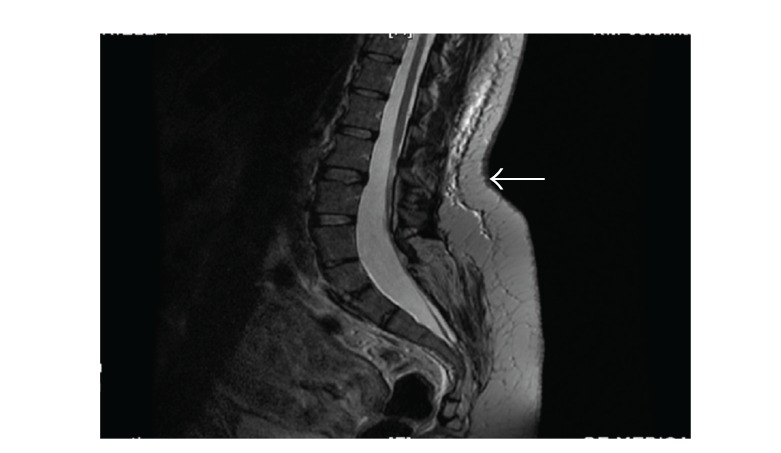
Sagittal view of the patient's MRI scan with spinal cord. The arrow approximately indicates puncture site.

**Figure 2:**
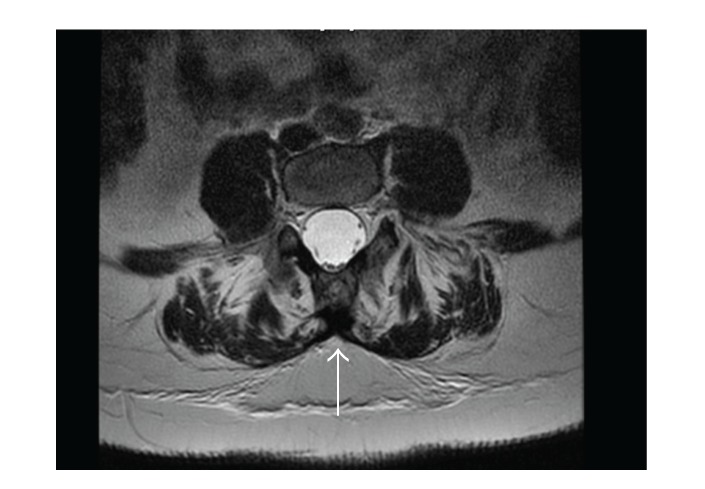
The patient's MRI transversal view: evidence of sacral bone interruption.

On the 4th postoperative day, our patient underwent MRI to precisely evaluate the anatomy of her vertebral column. MRI was performed with a 1.5 T scanner employing spin-echo and fast spin-echo sequences and showed interruption of posterior arches at the sacral level, without herniation of the meningeal sac, but with dural ectasia and marked atrophy of paravertebral muscles that had been almost completely replaced with fat. The medullar cone was located at the L2-L3 level, without any focal alterations of signal intensity. No abnormalities in the presacral subcutaneous fat were detected (Figures 1 and 2).

The patient and the baby left the hospital on postoperative day 5.

## 2. Discussion

Neural tube defects cover a wide range of structural anomalies, from anencephaly to myelomeningocele, meningocele, occult spinal dysraphism, and spina bifida occulta.

Spina bifida occulta is characterised by radiographic evidence of a lack of continuity in the lumbar or sacral vertebral posterior arches without clinical signs and symptoms of neurologic deficits [1]. It has a high frequency in the general population (about 20%) and usually carries no adjunctive risk related to neuraxial anaesthesia, which is commonly used in these patients without awareness of the existence of bony defects.

Our patient had occult spinal dysraphism, a condition in which neurologic signs or symptoms (the dysmorphic foot in this case) or dyschromic areas on the skin or hair puffs (this patient had both) are associated with lumbar or sacral posterior bony anomalies.

In occult spinal dysraphism and frank spina bifida, primary sources of concern regarding neuraxial anaesthesia are structural abnormalities, such as incomplete formation of bony laminae, abnormal spinal cord anatomy (splitting, as in dastematomyelia, or lower-than-usual position, as in tethered spinal cord), and vascular abnormalities, such as arteriovenous malformations or fistulae.

Vertebral anomalies, meningeal hernias, muscle atrophy, and fatty substitution of paravertebral tissues could make epidural space identification more difficult and increase the risk of an accidental dural wet tap [2, 3]. It is also possible that unexpected difficulties in epidural catheter placement can arise and that the spread of drug-containing fluid can be unpredictable [4]. In the case of reported anomalies of the spinal cord, the risk of spinal cord damage or intra-rachideal hematoma could be higher than in the general population [5].

In the present case, an MRI scan could not be performed before hospital admission in time to inform the anaesthesiologist's choices. Without a precise knowledge of the patient's anatomy, continuous epidural and spinal analgesia for labour were discouraged because of the higher-than-usual risk of complications and insufficient efficacy of the procedure. Remifentanil intravenous administration was selected because it allows avoidance of vertebral manipulation [6–10].

As evaluated by VAS score, remifentanil was effective in reducing pain throughout the almost 10-hour period of administration but showed reduced analgesic efficacy in late-stage labour when near-complete dilation was achieved.

At that point, the patient felt tired because of the labour and the 24-hour sleep deprivation; she could not fully cooperate with calling for a bolus at the onset of a uterine contraction to allow sufficient plasma drug concentration at the contraction peak. She was abruptly awakened by uterine contractions when they had already reached significant painful strength, and the bolus effect was inappropriately obtained only at the end of contraction. The need for coherent patient cooperation did not allow for rest and relaxation.

As in this case, after several hours of infusion, exhaustion and psychotropic drug activity may reduce the patient's cooperative ability. Tolerance or ultrafast addiction could also appear together with an increased drug need in second-stage labour. 

In the context of caesarean section, general anaesthesia risks are high and wellknown in obstetric patients (e.g., altered gastric emptying and a failed intubation incidence of about 1/300).

Even single-shot spinal anaesthesia complications might be increased by an altered anatomy of the vertebral column and neuraxis, but medical literature is rich in case reports of successful neuraxial anaesthetics, and no generally accepted recommendation exists about its avoidance in patients with neural tube defects. An absence of major motor or sensory signs and continence was against a diagnosis of tethered cord in our patient. For these reasons, we chose subarachnoid anaesthesia at the moment of operative delivery. 

*A posteriori* analysis, even in this uncomplicated case, suggests that a higher-level lumbar puncture could have been dangerous because of L2-L3 termination of the spinal cord, although it was not classified as a real “tethered cord.” Dural sac ectasia, with associated increased volume of cerebrospinal fluid, might have contributed to the nonimmediate onset and the short termination of spinal anaesthesia with a dilution effect.

MRI should be obtained prior to the final weeks of pregnancy in women with neurologic signs and symptoms to allow safer neuraxial procedures in case of emergent, full-stomach caesarean section [4].

## 3. Conclusions

Remifentanil intravenous infusion may represent a valid analgesic technique when the risk-benefit ratio of neuraxial anaesthesia appears unfavourable, but use of remifentanil requires full patient cooperation and assiduous monitoring.

Subarachnoid anaesthesia for caesarean section may be performed in patients with symptomatic spinal dysraphism, but to be low risk, the procedure will be better executed with precise knowledge of the patient's MRI anatomy.
